# ASSOCIATION OF PROCOLLAGEN TYPE III N-TERMINAL PEPTIDE WITH AGE AND MORTALITY: THE LONG LIFE FAMILY STUDY

**DOI:** 10.1093/geroni/igae098.2399

**Published:** 2024-12-31

**Authors:** Ryan Cvejkus, Mary Wojczynski, Shiow Jin, Bharat Thyagarajan, Joseph Zmuda

**Affiliations:** University of Pittsburgh, Pittsburgh, Pennsylvania, United States; Washington University in St. Louis, St. Louis, Missouri, United States; Washington University in St. Louis, St. Louis, Missouri, United States; University of Minnesota, Minneapolis, Minnesota, United States; University of Pittsburgh, Pittsburgh, Pennsylvania, United States

## Abstract

Procollagen type III N-terminal peptide (P3NP) is released into circulation during collagen biosynthesis and is a biomarker of fibrosis in several organs. Herein, we tested the association between baseline plasma P3NP concentration, measured using a sandwich enzyme-linked immunosorbent assay, and risk of all-cause mortality over 17 years in men (n=1882) and women (n=2277) aged 69.9 ± 15.5 (range, 32 – 110) years at baseline. Baseline median [IQR] P3NP level in the full cohort was 5.4 [4.5, 6.7] μg/L, and there was a significant positive linear trend across 10-year age groups. Among those aged 30-39, the median P3NP was 5.2 [4.4, 6.0] μg/L, and among centenarians, the median P3NP was 9.7 [7.9, 11.5] μg/L. Mortality risk, using 1442 observed deaths, was calculated per standard deviation (SD) of log P3NP using Cox Proportional Hazards models adjusted for baseline age, sex, height, weight, field center, alcohol use, smoking history, chronic diseases, serum creatinine, and familial relatedness. A 1 SD higher P3NP level was associated with an 18% increased risk of mortality (HR: 1.18, 95% CI: 1.10 - 1.26). The association was non-significantly stronger in women (HR: 1.25, 1.12 – 1.40) compared with men (HR: 1.11, 1.01 – 1.21). After further adjustment for physical function, the overall association remained but was attenuated (HR: 1.10, 1.02 – 1.18). In conclusion, higher P3NP was independently associated with mortality after adjustment for important covariates, especially in women, suggesting that the prevention of excess collagen deposition (fibrosis) may be particularly important for overall health and survival.

